# Inflammasome NLRP3 Potentially Links Obesity-Associated Low-Grade Systemic Inflammation and Insulin Resistance with Alzheimer’s Disease

**DOI:** 10.3390/ijms22115603

**Published:** 2021-05-25

**Authors:** Anna Litwiniuk, Wojciech Bik, Małgorzata Kalisz, Agnieszka Baranowska-Bik

**Affiliations:** 1 Department of Neuroendocrinology, Centre of Postgraduate Medical Education, Marymoncka 99/103, 01-813 Warsaw, Poland; alitwiniuk@cmkp.edu.pl (A.L.); wojtmed@wp.pl (W.B.); mkalisz@cmkp.edu.pl (M.K.); 2 Department of Endocrinology, Centre of Postgraduate Medical Education, Cegłowska 80, 01-809 Warsaw, Poland

**Keywords:** obesity, insulin resistance, inflammasome, Alzheimer’s disease

## Abstract

Alzheimer’s disease (AD) is the most common form of neurodegenerative dementia. Metabolic disorders including obesity and type 2 diabetes mellitus (T2DM) may stimulate amyloid β (Aβ) aggregate formation. AD, obesity, and T2DM share similar features such as chronic inflammation, increased oxidative stress, insulin resistance, and impaired energy metabolism. Adiposity is associated with the pro-inflammatory phenotype. Adiposity-related inflammatory factors lead to the formation of inflammasome complexes, which are responsible for the activation, maturation, and release of the pro-inflammatory cytokines including interleukin-1β (IL-1β) and interleukin-18 (IL-18). Activation of the inflammasome complex, particularly NLRP3, has a crucial role in obesity-induced inflammation, insulin resistance, and T2DM. The abnormal activation of the NLRP3 signaling pathway influences neuroinflammatory processes. NLRP3/IL-1β signaling could underlie the association between adiposity and cognitive impairment in humans. The review includes a broadened approach to the role of obesity-related diseases (obesity, low-grade chronic inflammation, type 2 diabetes, insulin resistance, and enhanced NLRP3 activity) in AD. Moreover, we also discuss the mechanisms by which the NLRP3 activation potentially links inflammation, peripheral and central insulin resistance, and metabolic changes with AD.

## 1. Introduction

Alzheimer’s disease (AD), believed to contribute to 60–70% of neurodegenerative dementia cases, is a complex disorder that develops gradually and progressively with symptom progression over time, from mild forgetfulness to severe mental impairment. According to the World Alzheimer Report, it is estimated that 50 million people worldwide have dementia, and the number of people with dementia is projected to increase to 82 million by 2030 and to 152 million by 2050 [1].

Although aging is the leading risk factor for the development of Alzheimer’s disease, growing evidence, also from animal models, indicates that metabolic dysfunctions may have a crucial role in the etiology of AD [2,3]. Obesity and type 2 diabetes mellitus (T2DM) are reported to be related to AD [3,4]. It has been hypothesized that CNS inflammation takes part in the progression of chronic neurodegenerative diseases, but the mechanisms are still unclear. It is also possible that T2DM, or even prediabetes, can modulate the expression of brain pro-inflammatory cytokines in AD [5]. Additionally, both prediabetes and T2DM promote microglia activation in the mice AD model, thus confirming that the inflammatory process may serve as a link between AD and T2DM [6].

Obesity, characterized by hypertrophy and hyperplasia of adipocytes, is accompanied by chronic local inflammation [7]. Excessive accumulation of fat leads to enhanced expression and release of pro-inflammatory cytokines including tumor necrosis factor α (TNFα), interleukin-6 (IL-6), adipokines, and monocyte chemoattractant protein-1 (MCP-1), which further recruit immune cells to intensify inflammation in adipose tissue [8,9]. Additionally, adipose tissue also contains numerous immune cells, and its total number increases dramatically with the grade of obesity. The downregulation of M2 macrophages with anti-inflammatory phenotype and the activation of M1 macrophages with pro-inflammatory phenotype can exaggerate inflammation and insulin resistance in adipocytes [10]. Innate immune cells such as macrophages can induce inflammatory reactions through detection of pathogen- or danger-associated molecular patterns (PAMPs or DAMPs) using a wide range of pattern-recognition receptors (PRRs) [11,12]. Adiposity-related inflammatory factors lead to the formation of inflammasome complexes. Inflammasomes are cytosolic multiprotein complexes that recognize both PAMPs and DAMPs. These high-molecular-weight factors are responsible for the activation, maturation, and release processes of the pro-inflammatory cytokines interleukin-1β (IL-1β) and interleukin-18 (IL-18). Moreover, obesity-related factors are important activators of inflammasome-derived cytokines [13,14].

Recent advances have highlighted that various pathways could be regulators of the pathological features in Alzheimer’s disease. We present current knowledge of the metabolic dysregulations, including the NLRP3 inflammasome activation, and their contribution to AD pathology. The review includes a broadened approach to the role of obesity-related diseases (obesity, low-grade chronic inflammation, type 2 diabetes, insulin resistance, and enhanced NLRP3 activity) in AD. Moreover, we also discuss the mechanisms by which the NLRP3 activation potentially links inflammation, peripheral and central insulin resistance, and metabolic changes with AD.

## 2. Alzheimer’s Disease

From the clinical point of view, AD is accompanied by a progressive decline in memory and executive functions, as well as impairment of daily living activities [15]. The first, early symptoms of AD have been associated with the loss of episodic memory and difficulties in learning new information. When AD progresses, there is greater memory loss, cognitive impairment, and behavioral change, along with dysfunction of language and speech [16,17].

There are numerous classifications of AD. The most common division based on the patient’s age of onset includes late-onset AD (after 65 years; LOAD) and early-onset AD (before 65 years; EOAD). It has also been described that in some, but not all, EOAD cases there is a family history of dementia, which is characterized as early-onset familial AD (EOFAD) [18]. EOFAD is associated with genetic mutations in the three main genes involved in the amyloid processing, including amyloid precursor protein (APP) and presenilin genes (PSEN1 and PSEN2), whose products participate in the processing of APP [19,20]. In addition to the above-mentioned mutations, there are also identified variants in ~20 other genes that increase the risk of AD slightly (i.e., <2%). These susceptibility genes play a key role in brain development, cytoskeletal organization, and immune function [18]. Nevertheless, it has been estimated that 25% of all AD is familial (i.e., ≥3 persons in a family have AD) [18].

Neuropathological lesions in AD are associated with multiple changes at the cellular level. The typical histopathological features include amyloid β (Aβ) formation and accumulation, mitochondrial damage, loss of synapses, activation of microglia (gliosis) and astrocytes (astrocytosis), phosphorylation of tau, and neurofibrillary tangles formation (NFT) [21]. All of these pathological processes lead to neuronal death, which is observed in the brains of AD patients. 

The metabolic dysfunction may stimulate the Aβ aggregate formation [4,22]. Furthermore, AD, obesity, and T2DM share similar risk factors and some clinical and biochemical features. These particular features are associated with chronic inflammation, increased oxidative stress, and impairment in insulin signaling and energy metabolism [23,24]. Additionally, due to the potential multifactorial role of obesity in pathological processes seen in AD, obesity could serve as a risk factor for this disease [25]. Of note, patients with T2DM have a 2-fold higher risk of developing AD than healthy subjects [26].

## 3. Insulin Signaling Pathway in the Brain

### 3.1. Insulin in the Brain

Insulin is a pancreatic hormone secreted to plasma in response to hyperglycemia. It is a key factor controlling blood glucose concentration. It facilitates cellular glucose uptake in the peripheral tissues (liver, adipose tissue, and skeletal muscles) by activating the phosphatidylinositol 3-kinase (PI3K)-protein kinase B (Akt) pathway [27]. Subsequently, the activated Akt kinase phosphorylates Akt substrate 160 kDa (AS160), which recruits GLUT4 (glucose transporter 4) to the plasma membrane, allowing glucose to efficiently enter the cells (adipocyte and muscle cells). Moreover, peripheral insulin is preferentially transported by the receptor-mediated mechanism to the brain through the blood–brain barrier (BBB). Moreover, our and other authors’ in vitro studies indicated that insulin can also be synthesized and released directly in the brain by neuronal and glial cells, including astrocytes [28,29]. Insulin and the PI3K-Akt signaling pathway play a crucial role in neuronal health and synapse formation and maintenance as well as could promote neurogenesis by modulating neuronal stem cell proliferation, differentiation, and survival [30,31].

### 3.2. Insulin Receptors

Insulin acts on target cells through a specific transmembrane insulin receptor (IR). The IR has the tyrosine kinase activity and is composed of α/β subunit dimers that are linked by disulfide bonds. Due to alternative splicing of the insulin receptor gene, two insulin receptor isoforms are found: insulin receptor A (IR-A) and insulin receptor B (IR-B) that differ from each other based upon the absence (IR-A) or presence (IR-B) of a 12-amino acid sequence. Additionally, some functional differences have been reported [32].

The IRs are abundantly expressed on almost all cell types of the brain and densely localized in synapses in the olfactory bulb, hypothalamus, hippocampus, cerebral cortex, and cerebellum. On the cellular level, astrocytes express both IR-A and IR-B, while neurons express exclusively the IR-A [33].

### 3.3. Insulin Signal Transduction

**Figure 1:**
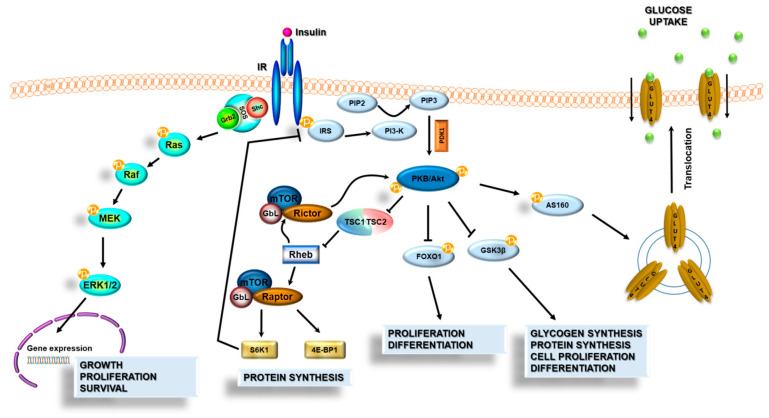
Scheme of the insulin signal transduction pathways. Insulin acts on target cells through its specific insulin membrane receptor (IR). IR consists of two α subunits located outside the cell membrane and two β subunits anchored to the cell membrane. Activation of the IR causes autophosphorylation of the β subunits, which recruit the next cellular proteins. Insulin activates two major signal transduction pathways: the PI3K-PKB/Akt-dependent pathway and the MAPK (mitogen-activated protein kinase) kinase pathway.

Under normal conditions, insulin binds to IR and activates two major signal transduction pathways: the previously mentioned PI3K-Akt-dependent pathway and the MAPK (mitogen-activated protein kinase) kinase pathway (Figure 1). Furthermore, insulin can bind not only to the classical forms of insulin receptor but can interact also with the homologous insulin-like growth factor-1 receptor (IGF-1R) or with insulin receptor–IGF-1R heterodimers. However, insulin interaction with IGF-1R has reduced affinity when compared to the classical insulin receptor [32].

The binding of insulin to IR triggers the phosphorylation and activation of insulin receptor substrate (IRS), which in turn forms a docking site for phosphatidylinositol 3-kinase (PI3K) at the cell membrane. Next, PI3K converts phosphatidylinositol 4,5-bisphosphate (PIP2) to phosphatidylinositol 3,4,5-trisphosphate (PIP3), a second messenger that activates phosphoinositide-dependent protein kinase 1 (PDK1), and recruits protein kinase B (PKB/Akt) to the cell membrane. Consequently, activated PKB/Akt controls many downstream pathways and regulates many processes (e.g., growth, proliferation, differentiation, survival cells, and metabolic processes). The Ras-mitogen-activated protein kinase pathway leads to the activation of genes that are involved in cell growth, proliferation, and survival [34].

Activation of the MAPK-dependent kinase pathway via receptor-activated Ras or Rheb (brain isoform) induces an increase in the transcription factor levels, such as c-Fos or c-Myc, which stimulates the growth of cells as well as their differentiation and survival [35]. Additionally, under conditions of oxidative stress, when neurons are structurally damaged and their proper functioning is impaired, the MAPK pathway can also induce apoptosis of the nerve cells. However, a study by Zakharova et al. showed that the insulin-induced activation of ERK1/2 at early stages of oxidative stress development appears to contribute in cortical neurons to the neuroprotective ability of insulin to promote neuronal viability and inhibition of the oxidative stress development in the cells [36]. 

### 3.4. The Role of AKT and Glycogen Synthase Kinase-3 (GSK-3) in AD

The neuroprotective effect of insulin in the brain is closely related to the activation of the PI3K-Akt pathway. Akt acts as a key player in insulin signaling and function. An increasing amount of data indicates that insulin activates Akt in cortical neurons [36,37]. Next, phosphorylated Akt phosphorylates and thereby inactivates both isoforms (α and β) of cytosolic glycogen synthase kinase-3 (GSK-3). Active GSK-3α isoform regulates the production of the β-amyloid peptide. In turn, the GSK-3β isoform is involved in the hyperphosphorylation of the tau protein [38]. It is worth highlighting that insulin administration downregulates the GSK-3β activity and consequently prevents Aβ intra-neuronal accumulation and reduces the tau protein phosphorylation. Moreover, Akt and GSK-3 seem to be crucial for modulation of the balance between LTP (long-term potentiation) and LTD (long-term depression) [39]. In addition, GSK-3β plays an important role in mitochondrial biogenesis, bioenergetics, permeability, and mitochondria-dependent apoptosis [40]. Insulin-activated Akt inhibits the expression of caspase-9 and Bcl-2 associated apoptotic factors, making it critical for cell survival [41]. 

## 4. Glucose Metabolism in the Brain

### 4.1. Physiological Brain Glucose Metabolism

Glucose is the main substrate for brain energy production in adults [42]. It is estimated that the adult brain consumes ~20% of glucose-derived energy [43]. Therefore, maintaining glucose homeostasis is of utmost importance for the proper functioning of this organ. Additionally, neurons have very high energy requirements. Therefore, neuronal activity relies heavily on mitochondrial function and oxidative phosphorylation that provide ATP for ion homeostasis necessary for synaptic transmission, action potential, and the recycling of neurotransmitters. Consequently, neurons are especially vulnerable to mitochondrial damage [44]. In addition to its use for ATP production, glucose also serves as the precursor for many compounds synthesized within the brain (e.g., neurotransmitters and neuromodulators), which are involved in the regulation of neuronal and glial cell function [45]. 

Glucose is transported into the cell across the cell membrane by specific sodium-independent facilitated glucose transporters (GLUTs). Among the 14 members of the GLUTs family, GLUT1, GLUT3, and GLUT4 are the major brain glucose transporters. GLUT1 is mainly located in the endothelial cells of the blood–brain barrier and astrocytes, whereas GLUT3 and GLUT4 are highly expressed in neurons. The intracellular glucose metabolism depends either on glycolysis and the pentose phosphate pathway in the cytoplasm or on the intracellular Krebs cycle and mitochondrial respiratory chain [46]. Glucose transport from the peripheral circulation across the BBB into the brain tissue and across the cell membrane into neurons are mainly insulin-independent processes involving GLUT1 in the BBB and GLUT3 in neurons [47]. However, the study by Pearson-Leary et al. indicated that insulin induces GLUT4 translocation to the neuron cell membrane by the Akt-dependent mechanism [48]. Moreover, it has been suggested that insulin-induced GLUT4 may improve glucose flux into neurons during periods of high metabolic demand, such as learning. 

### 4.2. Disturbed Glucose Metabolism in AD

An increasing number of studies indicate that sporadic AD etiopathogenesis may also be associated with dysfunctional brain insulin signaling and the subsequent disruption of glucose metabolism [49]. Interestingly, the positron emission tomography studies (^18^F deoxyglucose positron emission tomography; 18FDG-PET) showed a reduced brain glucose uptake in the hippocampus and brain cortex of familial and sporadic AD subjects [50,51]. Additionally, the reduction in glucose uptake in those specific regions was also related to the synaptic density and function, which suggested a direct correlation between the affected region and cognitive impairment [52]. However, it is still unclear whether abnormalities of brain glucose homeostasis in AD are related to peripheral glucose concentration. A clinical study by An et al. indicated that disturbances in brain glucose homeostasis are intrinsic to AD pathogenesis and may appear several years before the onset of clinical symptoms. In detail, the authors observed that the activity of three rate-controlling enzymes of glycolysis (hexokinase, phosphofructokinase, and pyruvate kinase) was significantly reduced in the inferior temporal gyrus (ITG) and the middle frontal gyrus (MFG) of patients suffering from AD. These findings were also associated with the greater severity of both neurofibrillary and Aβ pathology. Moreover, there is evidence that lower cerebral glucose utilization is associated with lower rates of glycolysis and higher brain tissue glucose concentrations, which may lead to decreased GLUT3 protein levels, especially in the brain regions vulnerable to AD pathology. Finally, higher concentrations of plasma fasting glucose measured before death as well as greater increases in fasting plasma glucose over time were associated with higher brain tissue glucose concentrations in the brains of individuals of AD [53]. These findings confirmed earlier epidemiological studies, which described the role of different markers of T2DM, including impaired fasting glucose, insulin resistance, and high glycated hemoglobin levels as predictors of increased incidence of dementia [54].

## 5. Insulin Resistance as a Common Factor Linking Obesity, T2DM, and AD

### 5.1. Malfunction of Insulin Signaling

Under physiological conditions, insulin plays an essential role in the brain. This peptide is of great importance, among others, in neuroprotection, regulation of selected neurotransmitter levels, neuronal plasticity, and cholinergic functions that are involved in learning and memory processes [3]. Therefore, disturbed brain insulin signaling can greatly affect cognitive impairment and neurodegeneration, particularly mild cognitive impairment and AD [55]. 

Interestingly, obesity, diabetes, and cognitive impairment/Alzheimer’s disease share in common a core feature that is target-organ insulin resistance [55]. Insulin resistance is defined as reduced tissue responsiveness to the physiological action of insulin [56]. Similarly, brain insulin resistance is an impairment of brain cells to respond to insulin input [57]. Furthermore, peripheral insulin resistance is associated with impaired brain insulin action [56]. Some obesity/T2DM-associated factors and mechanisms, related to inflammatory pathways, could modulate peripheral insulin signaling. Among them are intracellular lipid deposition/imbalance, mitochondrial dysfunction, hypoxia, increased oxidative stress, as well as enhanced levels of circulating cytokines (TNFα, IL-1β, IL-6, and IL-18), chemokines (MCP-1), pro-inflammatory adipokines, and serum fatty acids [30,31]. Noticeably, TNFα, IL-1β, IL-18, and IL-6 are the most important cytokines involved in the development of insulin resistance. Of note, disturbed immune response negatively affects organs, including the brain. Furthermore, metabolic dysfunction in T2D, obesity, and insulin resistance plays a role in promoting cognitive dysfunction and impacts the pathogenesis of AD [58]. 

**Figure 2:**
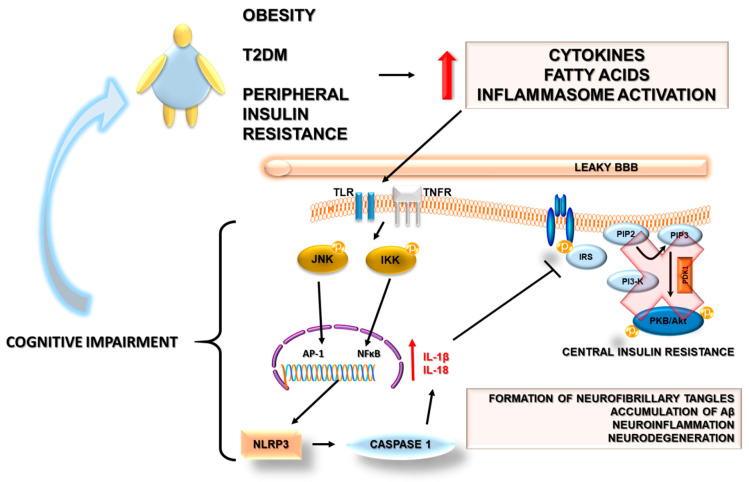
Potential mechanisms linking peripheral and brain insulin resistance developing during obesity, type 2 diabetes mellitus, and Alzheimer’s disease. Details in the text. The bold red arrow indicates an increase in the release of cytokines and fatty acids and an increase in inflammasome activation. The thin red arrow indicates an increase in the release of IL1β and IL-18.

Physiologically, activation of insulin receptors stimulates tyrosine phosphorylation of insulin receptor substrates-1 (IRS-1) to initiate downstream signaling pathways [59]. However, in the course of metabolic diseases related to obesity, both elevated cytokines (e.g., TNFα) and free fatty acid (FFA) trigger serine phosphorylation of IRS-1 by stress kinases, resulting in the development of insulin resistance [60] (Figure 2). 

The major intracellular stress kinase that links TNFα to inhibitory serine phosphorylation of IRS-1 in T2DM and obesity is the c-Jun N-terminal kinase (JNK) family of MAPK [49,61]. Moreover, the activation of the JNK may serve as a bridge between peripheral and central insulin resistance. Furthermore, it could also link insulin resistance with AD. Some stressor factors in AD including oxidative stress, Aβ accumulation, neurotrophic deprivation, and proinflammatory cytokines could activate the JNK pathway [62]. Of note, studies revealed that both oligomers of Aβ and a high-fat diet stimulated the JNK phosphorylation in the hippocampal neurons, leading to the downregulation of insulin signaling [63,64]. Deficiency in the insulin-IRS-PI3K-Akt signaling pathway could result in the activation of GSK3β, promotion of hyperphosphorylation of tau protein, and the occurrence of neuronal dysfunction [38]. Additionally, JNK plays a role in mediating degeneration and apoptosis in the brain as well as in impairing neuronal insulin axis physiology [49]. Further, postmortem brains in the AD model showed increased serine phosphorylation of IRS-1 as well as JNK expression [64]. This phenomenon was also observed in human postmortem brain samples, and additionally, its positive co-localization with Aβ was revealed [65]. 

Another stress kinase related to insulin resistance is IκBα kinase (IKK). IKK is activated both by TNFα in the case of peripheral insulin resistance and by Aβ in the brain, resulting in decreased neuronal insulin sensitivity [64,66]. 

### 5.2. Insulin Resistance and AD

Aβ metabolism is impacted by insulin and the threshold of insulin receptor sensitivity, whereas, conversely, Aβ interferes with insulin binding to its receptor and the expected biological response. Therefore, insulin resistance may not only be a risk factor for the development of AD but also may serve as a feature that aggravates its pathology [38,49]. A recent study revealed that in non-diabetic AD patients, peripheral insulin resistance was independently associated with reduced hippocampal glucose metabolism and with lower grey matter volume, confirming the hypothesis of insulin resistance impact on AD pathology [56]. Moreover, neuroimaging studies of the brain reported significant correlations between the presence of T2DM, obesity, and/or peripheral insulin resistance with decreased hippocampal volume [67,68]. Nevertheless, AD is a brain form of diabetes in which both ligand (insulin and IGF-1) deficiencies and receptor resistances coexist and mediate functional impairments in signaling pathways, and this pathology is named type 3 diabetes [69]. 

Based on the above data, it could be assumed that insulin resistance is exacerbated in metabolic diseases as well as in AD due to chronic inflammation. Noticeably peripheral insulin resistance found in T2DM and obesity could result in cognitive impairment by the development of central insulin resistance associated with neuroinflammation [70]. Neuroinflammation in combination with amyloid plaques results in synaptic dysfunction and neurotoxicity in age-associated dementia and AD [71]. Epidemiological studies support the association between cognitive dysfunction and diabetes, and consequently, in the diabetic population, the risk for all types of dementia is increased by 73% [49]. However, it should be noticed that there are still controversies concerning associations between diabetes and AD, as some researchers do not confirm this relationship [72]. On the other hand, many animal studies on the model of T2DM confirmed that T2DM promotes the development and accumulation of AD-associated pathologies, such as amyloid-β plaques, tau phosphorylation, and neurofibrillary lesions [73,74]

## 6. Inflammasomes as a Possible Link between Inflammation-Associated Peripheral and Brain Insulin Resistance in Metabolic Disease and AD

### 6.1. Inflammation and AD

Aging is the largest known risk factor for AD [75]. Undoubtedly, the process of aging is related to an age-related inflammatory response characterized by chronic, systemic inflammation named inflammaging [76]. Additionally, some modifiable risk factors for AD such as diabetes, obesity, hyperlipidemia, and hypertension also coexist with systemic inflammation [75]. In turn, central and systemic inflammation promotes AD progression and even initiates neurodegeneration [75].

Neuronal degeneration is associated with inflammatory processes in the CNS. Microglia play a pivotal role in inflammatory reaction. [77]. In particular, microgliosis and astrocytosis are often observed in the brains of AD patients. In the course of AD, microglia gather around the Aβ plaques [78] and induce massive neuronal cell death through secretion of TNFα, IL-1β, IL-18, IL-6, chemokines, neurotransmitters, reactive oxygen species (ROS), and nitric oxide (NO) [79]. Consequently, the hypothesis concerning neuroinflammation as a driver of neurodegeneration has been made. Potential implications of neuroinflammation for AD have been indicated in several genetic studies, neuroimaging evaluations, and biopsies of patients [80]. 

In addition, a possible link between central and systemic immune responses has also been indicated. In detail, neuroinflammation induces the efflux of the CNS proteins, such as Aβ or inflammatory mediators, across the blood–brain barrier. These processes may cause a systemic immune reaction and result in the recruitment of myeloid or lymphocytic cells in the CNS [81]. Moreover, under pathological conditions, the CNS can also be infiltrated by peripheral inflammatory cells. The loss of BBB integrity seen in AD in combination with the consequence of the migration of immune system cells in cerebral vessels may result in exacerbating the neurodegenerative process and associated inflammatory responses [82]. Noticeably, local and systemic inflammation induced by obesity or type 2 diabetes mellitus can increase BBB permeability by, among other factors, obesity-related NF-κB-mediated inflammation of the BBB via TLR4 receptor [83].

### 6.2. Adiposity-Related Inflammation 

An increasing amount of data suggests that adipose tissue-associated inflammation is the primary mechanism by which obesity becomes a risk factor for developing cognitive disorders, especially AD [25,84]. The presence of an adiposity-related inflammatory microenvironment leads to the formation of inflammasome complexes that may amplify innate immune response not only locally in adipose tissue but also in the whole organism, making it systemic [85,86]. On the other hand, it has been suggested that inflammasome contributes to the inflammaging, which likely represents the initial stages of cognitive dysfunction and neurodegeneration [76]. Moreover, increased inflammasome activity in adipose tissue has been proposed as an important mediator of obesity-induced systemic inflammation as well as a factor involved in the development of insulin resistance. Both of these pathological features are inherently associated with cognitive impairment and neurodegeneration [86]. Moreover, innate immunity-mediated neuroinflammation actively contributes to the onset and progression of neurodegenerative diseases, including AD. Further, inflammasomes are active players in the progression of the innate immune response [87].

### 6.3. The Role of Inflammasomes in Adiposity-Related Inflammation and Insulin Resistance

**Figure 3:**
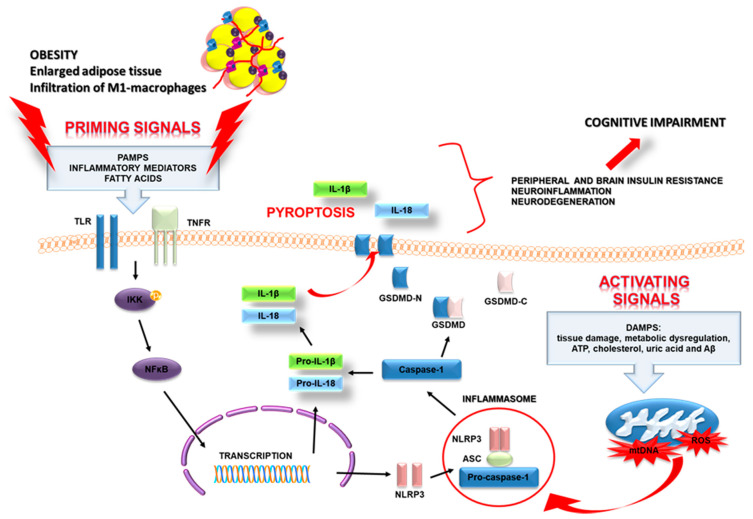
Scheme of the NLRP3 inflammasome activation in metabolic diseases. The activation of NLRP3 requires two distinct, independent signals. The first signal has been referred to as a “priming signal”, while the second is referred to as an “activating signal”. The priming signal is started when PAMPs and/or other inflammatory mediators (IL-1β and TNF-α) bind to their respective receptors (PRRs, IL-1βR, and TNFαR). Binding to the receptors induces the activation and translocation into the nucleus of nuclear factor kappa-light-chain-enhancer of activated β cells (NF-κβ), which in turn, promotes the transcription of NLRP3, pro-IL-1β, and pro-interleukin-18 (pro-IL-18). The second signal activation of the NLRP3 complex involves several extracellular stimuli, DAMPS, such as tissue damage, metabolic dysregulation, ATP, cholesterol, uric acid, and amyloid β. It results in mitochondrial dysfunction and the release of ROS (reactive oxygen species) and mitochondrial DNA (mtDNA). ROS and oxidized mtDNA trigger NLRP3 inflammasome formation and activation, which allow caspase-1 to start the cleavage of pro-IL-1β and pro-IL-18. The active caspase-1 also cleaves the inactive pro-form of gasdermin D (GSDMD) into two fragments: the N-terminal domain and the C-terminal domain. The GSDMD N-terminal fragments are needed for pore formation on the cell membranes. These pores are necessary for the release of inflammatory factors (IL-1β, IL-18) and for cell swelling, and membrane rupture. They eventually trigger a lytic, pro-inflammatory form of cell death, termed pyroptosis. Moreover, the release of IL-1β and IL-18 may amplify innate immune response not only locally in adipose tissue but also in the whole organism, making it systemic. Consequently, it contributes to inflammaging, which likely represents the initial stage of cognitive impairment and neurodegeneration.

Inflammasomes are multiprotein complexes with the ability to stimulate innate immune responses. This response is induced by sensing pathogen- or danger-associated molecular patterns (PAMPs or DAMPs) that interact with a wide range of pattern-recognition receptors (PRRs) [11,12,88]. The PRRs include toll-like receptors (TLRs), retinoic acid-inducible gene I-like helicases (RLHs), and nucleotide-binding oligomerization domain-like receptors (NOD-like receptors; NLRs) [89,90]. The last one possesses a wide spectrum of recognition ability, not only of microbial structures but also of non-infectious factors related to tissue damage. The family of NLRs in humans comprises at least 23 different proteins having a similar domain arrangement [91]. The best-characterized inflammasomes are NLRP1 (nucleotide-binding oligomerization domain (NOD)-like receptor protein 1), NLRP2 (NOD-like receptor protein 2), NLRP3 (NOD-like receptor protein 3), NLRC4 (CARD domain-containing protein 4, also called IPAF (ICE-protease activating factor)), and AIM 2 (absent in melanoma 2) inflammasome [92]. Inflammasomes are present in the cytoplasm of many types of cells, not only in immune cells (such as macrophages, dendritic cells, T cells, and B cells) but also in neuronal cells, glial cells (microglia and astrocytes), and endothelial cells [93,94,95]. These high-molecular-weight platforms are responsible for the activation, maturation, and release processes of the pro-inflammatory cytokines IL-1β and IL-18 (Figure 3). 

There are two types of signaling pathways that activate the NLRP3 inflammasome: the canonical and noncanonical signaling pathway [96]. The canonical pathway depends on caspase-1 and involves inflammasome complexes detecting pattern PRR proteins and inducing recruitment of procaspase-1. The noncanonical signal pathway is mainly dependent on mouse caspase-11 or human caspase-4 and caspase-5. The noncanonical inflammasome is activated by lipopolysaccharide (LPS) [87]. 

Findings are suggesting that activation of the inflammasome complex (particularly NLRP3) by DAMPs has a crucial role in obesity-induced inflammation, insulin resistance, and T2DM. In 2010, Zhou and colleagues were the first researchers who indicated that the NLRP3 inflammasome may impact the regulation of adiposity and insulin sensitivity in the course of obesity [97]. This hypothesis was confirmed in further studies on NLRP3-lacking mice models [98,99]. Summarizing, the results may indicate the relationship between inflammasome activation and obesity-related systemic inflammation. Consequently, this phenomenon may be associated with the reduction of inflammasome-related cytokines in NLRP3-lacking mice as compared to the wild-type control. Furthermore, ablation of the *Nlrp3* gene also had a protective effect that prevented the obesity-induced activation of macrophages in adipose tissue (ATM) with M1 pro-inflammatory-like macrophage gene expressions, such as TNFα, chemokine ligand 20, and chemokine ligand 11, and that increased expression of M2 anti-inflammatory-like cytokines (interleukin-10). 

### 6.4. Inflammasomes and Metabolic Syndrome

Nonetheless, an issue of what kind of immunocompetent cell types are associated with inflammasome activation in human adipose tissue is still under intensive research. Pahwa et al., in an immunohistochemical analysis, observed that in human subcutaneous adipose tissue (SAT) expression of caspase-1 significantly correlated with mast cell and eosinophil density, while SAT expression of IL-1β and IL-18 showed correlation with mast cells abundance only. Surprisingly, the authors described that caspase-1, IL-1β, and IL-18 did not show any interconnection with macrophage density in SAT of patients with metabolic syndrome (MetS) [100]. On the contrary, the team of Kursawe reported that expression of NLRP3 related genes (TLR4, NLRP3, IL-1β, and caspase-1) and macrophage infiltration in abdominal SAT was increased in obese adolescents with high visceral/subcutaneous fat depots compared with BMI (body mass index)-matched subjects with a low visceral/subcutaneous fat amount. Moreover, increased expression of the inflammasome components CASP1 and macrophage marker CD68 was markedly correlated with visceral/subcutaneous fat distribution, which was, not surprisingly, inversely associated with insulin sensitivity [101]. Gao and colleagues, in in vitro models of human macrophages and primary fat cells, observed that macrophage-derived IL-1β reduced protein abundance of insulin signaling molecules, including IRS-1, PI3K p85α, GLUT4, and insulin-stimulated phosphorylation of Akt in human adipocytes [102]. These results are in agreement with a previously published study indicating that IL-1β is a key regulator of the translation of obesity-associated inflammation into insulin resistance in rodent models [103]. On the other hand, the novel findings of Chang and co-workers showed that insulin serves as a negative regulator of NLRP3 inflammasome activation in the course of severe LPS-induced inflammation [104].

The expression of the different components of NLRP3 inflammasome in adipose tissue of obese individuals with type 2 diabetes mellitus is directly associated with body weight as well as with the severity of T2DM [98]. Additionally, weight loss in diabetic patients correlated with a significant downregulation in *NLRP3* and *IL-1β* gene expression in subcutaneous adipose tissue. Very recently, Pahwa and colleagues made the novel observation that expression of caspase-1, as well as its main effectors IL-1β and IL-18, are significantly higher in SAT of patients with MetS compared with the matched healthy controls. This team observed that caspase-1 positively correlated with SAT IL-1β but not with IL-18 [100]. The lack of correlation with IL-18 could suggest that induction of IL-18 in SAT is not dependent on NLRP3 activation only but might be also driven by NLRP1 inflammasome, which mainly induces the production of IL-18 in adipose tissue [105,106]. Moreover, Jialal et al. indicated that in comparison with controls, the monocytes of MetS patients have increased TLR2 and TLR4 expressions as well as nuclear phosphorylation-p65 (NFκB) levels. Additionally, the authors observed a positive correlation between TLR4 and nuclear phospho-p65. These results further underscored that activation of TLRs, in turn, activates the intracellular NLRP3 signaling pathway in adipose tissue of humans [107]. A detailed scheme of the NLRP3 inflammasome activation is shown in Figure 3. 

Another study with microarray transcriptome analysis in SAT adipocytes and stromal vascular fraction (SVF) samples identified that the expression of NLRP3-related genes such as *NLRP3*, *PYCARD*, *IL-1β*, and *IL-18* was increased in adipocytes but not in the SVF samples of obese postmenopausal women when compared with controls [108]. The above data indicated that the aberrant chronic activation of the NLRP3 inflammasome by metabolic “danger signals” may be considered as a highly important factor in obesity-associated chronic diseases.

### 6.5. NLRP3 Inflammasome and Aging

Aging implies changes in the whole organism, including the immune system. The impairment of the immune system includes age-dependent thymic atrophy, dysregulation of T cell homeostasis, age-associated B cell expansion in adipose tissues, and overall pro-inflammatory phenotype [109,110]. Moreover, the whole cellular environment is influenced by senescence. In detail, it is modulated by the secretion of proinflammatory cytokines, proteases, and chemokines that lead to senescence-associated secretory phenotype (SASP) [85]. Chronic, low-grade inflammation that accompanies aging is termed inflammaging [111]. Its occurrence is linked with the deterioration of many diseases as well as exacerbating processes associated with age. Noticeably, excess nutrient availability along with metabolic disorders induce an inflammatory metabolic state named metaflammation [111,112]. Furthermore, increased systemic inflammation concomitant with metabolic alterations including obesity, insulin resistance, and dyslipidemia, may result in shortening lifespan and health span [113]. 

The role of the NLRP3 inflammasome activation in inflammaging and metaflammation should be highlighted. First of all, the aberrant activity of the NLRP3 inflammasome promotes the pathogenesis of diabetes and obesity that is related to insulin resistance. On one hand, the high-fat diet, obesity, and insulin resistance activate the NLRP3 inflammasome, while conversely, NLRP3-related processes induce the progression of obesity and insulin resistance by IL-1β -dependent impairment of pancreatic beta-cell and adipocyte function, and decreased insulin sensitivity [97,111,114]. The significance of the NLRP3 inflammasome activation in disturbed carbohydrate management was confirmed in animal models. The deletion of NLRP3 or inhibition of caspase-1 in mice resulted in improved insulin sensitivity and ameliorated obesity-associated pathologies [98,111,114]. Moreover, decreased systemic inflammation and reduced immune cell activation were seen in NLRP3 deficiency [112,114]. Importantly, elimination of the inflammasome components (NLRP3, ASC, and caspase-1) protect against T2DM [112,115]. 

Atherosclerosis is commonly associated with diabetes, obesity, and aging. Chronic NLRP3 inflammasome activity worsens the course of atherosclerosis [111]. However, atherogenic factors serve as activators of the NRLP3 inflammasome pathway [111,116]. Elimination or inhibition of the NLRP3 inflammasome complex, including NLRP3, ASC, or IL-1β, displays a protective function ameliorating inflammatory responses and atherosclerosis progression. NLRP3 deficiency was also found to improve lipid metabolism and to decrease inflammation, pyroptosis, and infiltration of more immune cells into plaques [111]. 

Aging is the main cause of cardiovascular diseases in healthy people [117]. Therefore, pathological processes aggravating age-related changes may influence the cardiovascular system. Interestingly, both NLRP3 and IL-1β have been proposed as new cardiovascular risk markers [118,119]. Moreover, an animal study conducted on NLRP3 −/− mice indicated enhanced inflammatory markers, but cardiac aging was prevented by the NLRP3 ablation, thus confirming that some heart disorders may be related to the NLRP3 complexes [117].

NLRP3 activity has also been shown to influence the immune system. It promotes the aging of the thymus that is accelerated by obesity [109,120]. Studies on the animal model indicated that NLRP3 or ASC ablation attenuated age-related thymic atrophy and promoted T cell diversity [120]. These results confirm the participation of NLRP3 in immunosenescence.

Nevertheless, the appropriate inflammasome response is critical for defense against infection [111]. Both excessive inflammasome activation and decreased NLRP3 inflammasome signaling may potentially result in a higher risk of death [111]. Indeed, mice without the NLRP3 pathway or ASC are more susceptible to bacterial infection, while excessive inflammasome activation, leading to exaggerated caspase-1 activation, IL-1β release, and pyroptosis, is related to severe cell and tissue damage and organ dysfunction, and ultimately to death [111,121,122] .

Of note, defective autophagy is seen in the elderly and promotes the activity of NLPR3 complexes [123]. In turn, inhibition of NLRP3 inflammasome enhanced autophagy [123]. Moreover, age-related activation of the NLRP3 inflammasome was found in aging hematopoietic stem cells and was caused by reduction of one of the sirtuins’ expression (SIRT2) and increased mitochondrial stress [124].

It is accepted that activation of the NLRP3 inflammasome promotes age-dependent systemic low-grade inflammation not only in the periphery but also in the brain, which as a result accelerates age-related neurodegenerative changes [111,125]. In addition, an animal study indicated that old mice without IL-1 receptors had improved measures of functional decline when compared with age-matched wild type [125]. 

In addition to NLRP3′s implications in metabolic and immune dysfunctions in the elderly, it is worth mentioning other roles of this inflammasome in decreasing longevity. As mentioned before, inflammaging and metaflammation, in which the NLRP3 inflammasome is an active player, could negatively influence the lifespan. Results from the experimental study confirm the contribution of the NLRP3 inflammasome in shortening length of life. Noticeably, ablation of NLRP3 resulted in several metabolic changes, including an increase in glucose tolerance, modulation of adipokine levels, and regulation of dyslipidemia. These findings were associated with common pathways, such as IGF-1, PI3K/AKT/mTOR, autophagy, and intracellular NAD+ levels. Moreover, the lack of NLRP3 in old mice was associated with lower IGF-1 concentrations [117]. As reported by Finkel, low serum levels of IGF-1 are the end product of decreased insulin/IGF-1 signaling, which is known to prolong life [113].

All the above-mentioned findings and observations support the thesis that the NLRP3 inflammasome is closely linked with inflammation and metabolic alterations in aging. 

### 6.6. Inflammasomes and AD

Importantly, the enhanced NLRP3 signaling pathway also has implications in neuroinflammatory processes [126,127]. Moreover, increasing lines of evidence from both animal models and clinical studies show that activation of NLRP3 is linked to pathogenic mechanisms in AD. Interestingly, the *Nlrp3-*null mutation protected against cognitive deficits in aged mice and mouse models of AD [125,128]. Administration of NLRP3 or caspase-1 inhibitors resulted in a significant increase of microglia ability to clear Aβ deposits, as well as in reduced Aβ deposition and improvement in cognitive impairment and hyperactive behavior [128,129].

Although the inflammasome signaling in the CNS is mainly attributed to microglia, the key innate immune cells of the brain, expression of inflammasome components have also been found in other cell types of the CNS, including neurons, astrocytes, perivascular CNS macrophages, oligodendrocytes, and endothelial cells [130,131,132,133]. Additionally, gene expression analysis of cultured PBMCs (peripheral blood mononuclear cells) from AD patients revealed a higher expression of NLRP3, ASC (adaptor apoptosis-associated speck-like protein containing a CARD), caspase-1, and caspase-5 as well as cytokines IL-1β and IL-18 [134]. 

**Figure 4:**
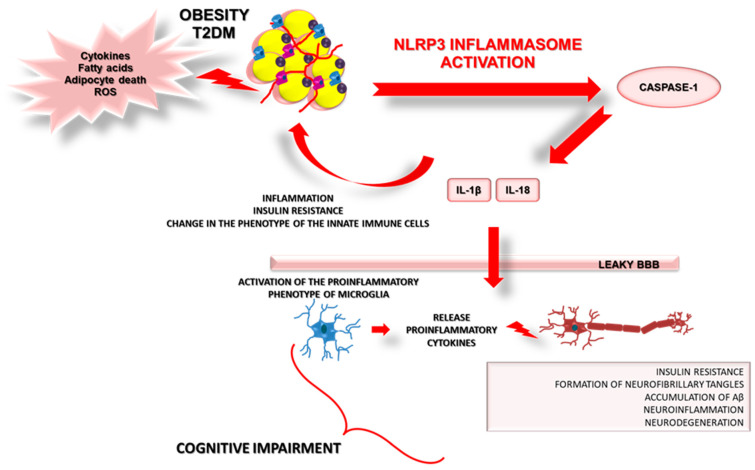
Potential mechanisms linking inflammation with obesity, NLRP3 inflammasome pathway, peripheral and central insulin resistance, and Alzheimer’s Disease. Details in the text.

Potential mechanisms linking inflammation with obesity, the NLRP3 inflammasome pathway, peripheral and central insulin resistance, and Alzheimer’s Disease are presented in Figure 4.

### 6.7. Peripheral Inflammasome Activation and Microglia

There is an open question regarding which mechanism is responsible for the impact of obesity-associated peripheral metabolic dysfunction and inflammation on brain processes. It could be related to the leakage of the BBB, by which obesity-associated DAMPs and/or PAMPs can easily access the brain and activate microglia to release pro-inflammatory molecules such as IL-1β and IL-18. Recently, Guo and colleagues hypothesized that NLRP3 induction in visceral adipose tissue (VAT) could initiate central microglial activation and cognitive impairment by increasing IL-1β. The authors used a series of dietary obesity and VAT transplantation experiments using Nlrp3−/− mutant mice and Tg mice with inducible deletion of IL1r1 (interleukin 1 receptor type 1) in CX3CR1(CX3C chemokine receptor 1)-expressing cells to verify their hypothesis. First, they observed that Nlrp3−/− mutant mice presented a protective phenotype against obesity-induced neuroinflammation. The NLRP3 lacking mice had a reduced level of IL-1β not only in VAT but also in the hippocampal lysates. Next, the authors indicated that transplantation of VAT from a wild-type donor increased expression of hippocampal IL-1β and influenced impaired cognition. However, VAT transplants from comparably obese Nlrp3−/− mutant donors did not cause any effect [86]. Finally, these data indicate that visceral adipose tissue NLRP3 inflammasome can impair memory via IL-1-mediated microglial activation. A suggestion that could be driven from this study is that NLRP3/IL-1β signaling could underlie correlations between visceral adiposity and cognitive impairment not only in mice but also in humans [86].

Data from studies concerning the NLRP3 inflammasome activity in metabolic diseases and AD are presented in Table 1.

### 6.8. AD Pathology Stimulates Central NLRP3 Activation

Studies based on clinical and animal experiments revealed that Aβ deposits could be involved in inflammasome stimulation in AD [87]. The increased expression of NLRP3, caspase-1, and IL-18 in the brains of AD patients was already described [134,141]. An animal study by the Griffin group showed that high levels of IL-1β in the brain induced tau protein hyperphosphorylation and neuronal damage [138]. In turn, accumulation and deposition of Aβ as well as NFT formation caused the release of mature IL-1β via activation of NLRP3 in microglia [136,142,143]. Therefore, overexpression of IL-1β may aggravate the central chronic inflammatory response. On the other hand, in AD, microglial excessive NLRP3 activation and elevated IL-1β concentration can exacerbate tau hyperphosphorylation, neurofibrillary tangles formation, and synaptic dysfunction induced by a detrimental chronic inflammation [144]. Moreover, NLRP3 activated by Aβ can induce enhanced production of IL-1β, promote microglial synthesis, and release proinflammatory cytokines and neurotoxic factors [128]. Therefore, it could be stated that the interrelation between NLRP3 and AD pathology is a vicious cycle.

### 6.9. The Possibilities of Pharmacological Modulation of NLRP3 Inflammasome Activity

NLRP3 inflammasome has become a promising drug target for inflammatory diseases. Possibly, inhibition of the NLRP3 inflammasome activity and the subsequent reduction of IL-1β and IL-18 production may change the phenotype of the innate immune cells and microglia in the brain. Pharmacological inhibition of the NLRP3 inflammasome may include, e.g., inhibition of NLRP3 inflammasome activation and formation, suppression of upstream signals, inhibition of caspase-1 activation, blockage of gasdermin D pore-forming protein (GSDMD) cleavage, and neutralization of IL-1β and IL-18 production [145]. The following examples present selected pharmacological possibilities for the prevention or treatment of AD.

The first report that selective pharmacological inhibition of the NLRP3 inflammasome is possible appeared in 2009 [146]. Lamkanfi et al. described that the sulfonylurea-containing compound glyburide inhibited NLRP3-induced pyroptosis and IL-1β secretion in murine and human macrophages. Glyburide is a small-molecule inhibitor commonly used as a treatment for T2DM. 

In 2019, Kuwar and colleagues developed a novel small molecule, JC124, through structural optimization of glyburide [147]. They observed that treatment with JC124 significantly decreased the expression of NLRP3, ASC, caspase-1, pro-IL-1β, TNFα, and inducible nitric oxide synthase (iNOS) in a rat model of traumatic brain injury [147]. Furthermore, beneficial effects of JC-124 as a specific NLRP3 inflammasome inhibitor were also described in a mouse model of AD by Yin et al. [148]. They observed that JC-124 attenuation of AD-associated features such as reduced amyloid pathology, reduced microgliosis and oxidative stress, and increased synaptic markers and astrocytosis [148].

MCC950, a diarylsulfonylurea-containing compound, is another selective inhibitor of the NLRP3 inflammasome. MCC950 inhibits both the canonical and noncanonical NLRP3 inflammasome activation and IL-1β production by abrogating ASC oligomerization [149]. In 2018, Qi and colleagues described that MCC950 treatment reversibly reversed the inhibition of long-term potentiation and improved synaptic plasticity deficits in an Alzheimer’s disease rat model [150]. Li et al. reported that MCC950 administration improved the spatial memory ability and brain histological morphology of senescence-accelerated mouse prone 8 (SAMP8 mice) and reduced the deposition of amyloid-β in mouse brain [151]. They also showed that the MCC950 inhibited the overexpression of NLRP3, caspase-1, and GSDMD in SAMP8 mouse neurons. Other authors found that in aged Alzheimer’s mice, treatment with MCC950 improved cognition and reduced Aβ, IL-1β, and microglial activation [129]. More studies are needed to warrant the exact potential of MCC950.

In 2018, Marchetti et al. described OLT1177 (rINN: dapansutrile) as a small molecule that specifically targets the NLRP3 inflammasome and prevents the activation of caspase-1 and the maturation and release of IL-1β [152]. Lonnemann et al. provided strong evidence that OLT1177 can ameliorate neurodegeneration and loss of synaptic plasticity in a murine AD model [153]. OLT1177 should be further explored in a clinical study in subjects with AD.

The role of PYD-only protein (POP)1 and POP2 in the inhibition of inflammasome activation cannot be omitted. These proteins directly interact with ASC, which are necessary for inflammasome function and block PYD interactions between NLRP3 and ASC. Moreover, POP1 and POP2 can suppress NF-κB activation, resulting in inhibition of the initial signal for inflammasome activation [154]. 

Interestingly, selected nonsteroidal anti-inflammatory drugs (NSAIDs), e.g., flufenamic acid, meclofenamic acid, and mefenamic acid, are effective and selective inhibitors of the NLRP3 inflammasome and show therapeutic effects on amyloid beta-induced memory loss in the transgenic mouse model of Alzheimer’s disease [155]. Therefore, the pathological features of AD might be reduced by the anti-inflammatory action of selected NSAIDs that could influence NLRP3 inflammasome activity [154].

The group of Shippy indicated that one of the ketone bodies, β-hydroxybutyrate (BHB), in a mice model of AD inhibited NLRP3 inflammasome activation and attenuated AD pathology by reduced Asc speck formation, plaque nucleation, and inflammation [156]. These findings explain at least partially the mechanisms of beneficial effects of a ketogenic diet or ketogenic supplements in AD. The favorable aspects of ketosis in both pathophysiological and clinical outcomes in AD were thoroughly reviewed by Lilamand and co-workers [157]. 

In addition to the above-mentioned compounds, other potential pharmacological inhibitors of NLRP3 inflammasome activation were presented by the group of Wani [158].

Additionally, a wide range of nutraceuticals may have clinical potential for suppressing inflammasome activity. Several nutraceuticals may also suppress the contribution of NLRP3 inflammasomes to many inflammation-linked pathologies. Amongst them are lipoic acid, ferulic acid, melatonin, phycocyanobilin, berberine, N-acetylcysteine, glucosamine, taurine, folate, vitamin B12, and betaine [159]. There are also reports suggesting that some plant-derived compounds and Chinese herbal medicines have a neuroprotective effect in treating AD by attenuating the activation of the NLRP3 inflammasome pathway [160].

Of note, a non-pharmacological approach to inhibit NLPR3 activation should also be considered. Data from the literature indicate a positive role of physical exercise in the suppression of NLRP3. Studies on animal models indicate that physical activity prevents Aβ-induced disturbances in the NLRP3 inflammasome pathway in the hippocampus of mice as well as reduces β-amyloid deposition by regulating NLRP3 inflammasome-controlled microglial phagocytosis [161,162]. In addition, a low-calorie diet may also upregulate pathways that inhibit NLRP3 activation [163]. Cellular metabolites, carbohydrates, and lipids can act as regulators of the NLRP3 inflammasome [158]. Therefore, modulation of the NLRP3 inflammasome by diet with reduced fat and carbohydrate intake should be taken under consideration. 

## 7. Conclusions

The above evidence clearly shows that obesity-related inflammation initially located in adipose tissue may finally have systemic effects on other organs and systems, including the CNS. As it has been shown, inflammasomes can act as a link between obesity, insulin resistance, and the development of neuroinflammation in neurodegenerative diseases, including AD. Although the adiposity-related mechanism of inflammasome activation is still unclear, inflammasomes may be a therapeutic target in the treatment of obesity. Further studies on inflammasomes could result in the development of innovative precision medicine approaches for the management of obesity and its complications, including those of neurodegenerative origin. The problem that should be considered when discussing inflammasome-targeted treatment is the multitude of stimulant compounds and, in addition, many physiological as well as pathological results of NLRP3 activity. The inhibition of NLRP3 should be balanced to avoid any other side effects. Unfortunately, to date, there is no NLRP3 inflammasome-targeted drug admitted to treatment. However, it should be highlighted that prevention and proper treatment of obesity and obesity-related diseases might lower the risk of AD. Finally, exercise and a low caloric/low-fat diet are a must for all overweight/obese patients. 

## Figures and Tables

**Table 1:** Findings from clinical studies and animal models concerning NLRP3 activity in metabolic diseases and AD. VAT, visceral adipose tissue; SAT, subcutaneous adipose tissue; ASC, adipose-derived stem cells; PBMC, peripheral blood mononuclear cell; STZ, streptozotocin.

Type of Study	Samples	Results	Reference
**Clinical study**	VAT	↑ expression of NLRP3, NLRP6, and ASC mRNA levels as well as the expression and release of IL-1β and IL-18 in VAT in patients with obesity and obesity-associated T2DM	[106]
	SAT	↑ expression of NLRP3, PYCARD, IL-1β, and IL-18 in adipocytes of obese postmenopausal women	[108]
	SAT	↑ genes expression of TLR4, NLRP3, IL-1β, and caspase-1 in obese adolescents with high VAT/SAT fat depot	[101]
	ASCs	↑ NLRP3 expression in ASCs from obese and/or T2DM patients	[135]
	SAT	↑ expression of caspase-1, IL-1β, and IL-18 in SAT from MetS patients	[100]
	PBMC	↑ genes expression of NLRP1, NLRP3, PYCARD, caspase-1, -5, -8, and downstream effectors IL-1β and IL-18 in monocytes from severe and mild AD	[134]
	The cortex of frontotemporal dementia (FTD) and AD patients	Elevated cleavage of caspase-1 and increased ASC levels and mature IL-1β	[136]
	The cortex of postmortem human brain of AD patients	↑expression of cleaved caspase-1 and IL-1β in the cortex of AD brains	[137]
**Animal study**	Old NLRP3−/− mice	Absence of NLRP3 diminished metabolic impairment induced by HFD during aging.	[99]
	Nlrp3−/− mutant mice and Tg mice with inducible deletion of Il1r1 in CX3CR1-expressing cells	Nlrp3−/− mutant mice indicate a protective effect against obesity-induced neuroinflammation.Reduced level of IL-1β in VAT and hippocampal lysates in Nlrp3−/− mutant mice.	[86]
	Primary cultures of microglia and cortical neurons from Spraque-Dawley rats	IL-1β induces tau protein hyperphosphorylation.	[138]
	Female C57BL/6JRccHsd and C57BL/6JRccHsd aged mice	Aged mice show caspase-1 activation in myeloid cells within the adipose tissue, as well as enhanced serum IL-18 and impaired glucose tolerance.	[139]
	Wildtype and APP/PS1 mice	Inhibition of NLRP3 reduces Aβ accumulation in APP/PS1 mice.	[129]
	Caspase11−/−, Nlrp3−/−, Asc−/−, and IL1r−/− mice	Age-associated enrichment of NFκB, IL-1, and IL-8 signaling pro-inflammatory pathways was partially dependent on the Nlrp3 inflammasome.	[125]
	Mouse model of sporadic Alzheimer’s disease (SAD) induced by STZ	Increased levels of NLRP3 in the cortex and hippocampus in the STZ group compared with those in the sham group.	[140]

## Abbreviations

18FDG-PET	^18^F deoxyglucose positron emission tomography
AD	Alzheimer’s disease
AIM 2	Absent in melanoma 2
Akt	Protein kinase B
AMPK	5’AMP-activated protein kinase
APP	Amyloid precursor protein
AS160	Akt substrate 160
Aβ	Amyloid β
BBB	Blood–brain barrier
Bcl-2	B-cell lymphoma 2
BMI	Body mass index
CARD	Caspase recruitment domain
CNS	Central nervous system
CX3CR1	CX3C chemokine receptor 1
DAMPs	Danger-associated molecular patterns
EOAD	Early-onset AD
FAD	Familial type of AD
FFA	Free fatty acid
GLUT1	Glucose transporter 1
GLUT3	Glucose transporter 3
GLUT4	Glucose transporter 4
GSDMD	Gasdermin D
GSK-3α	Glycogen synthase kinase-3α
GSK-3β	Glycogen synthase kinase-3β
HFD	High-fat diet
IDF	International Diabetes Federation
IGF-1R	Insulin-like growth factor 1 receptor
IKK	IκBα kinase
IL-10	Interleukin-10
IL-18	Interleukin-18
IL1r1	Interleukin 1 Receptor Type 1
IL-1β	Interleukin-1β
IL-6	Interleukin-6
IR	Insulin receptor
IRS	Insulin receptor substrate
ITG	Inferior temporal gyrus
JNK	c-Jun N-terminal kinase
LOAD	Late-onset AD
LRR	Leucine-rich repeats domain
LTD	Long-term depression
LTP	Long-term potentiation
MAPK	Mitogen-activated protein kinase
MCP-1	Monocyte chemoattractant protein 1
MetS	Metabolic syndrome
MFG	Middle frontal gyrus
MS	Multiple Sclerosis
mtDNA	Mitochondrial DNA
NBD	Nucleotide-binding oligomerization domain
NFT	Neurofibrillary tangles formation
NF-κβ	Nuclear factor kappa-light-chain-enhancer of activated β cells
NLRC4	CARD domain-containing protein 4, also called ICE-protease activating factor (IPAF)
NLRP1	NOD-like receptor protein 1
NLRP2	NOD-like receptor protein 2
NLRP3	NOD-like receptor protein 3
NLRP3	Nucleotide-binding oligomerization domain (NOD)-, leucine-rich repeat- (LRR)-, and pyrin domain-containing protein 3 (NLRP3) Inflammasome
NLRs	Nucleotide-binding oligomerization domain-like receptors (NOD-like receptors)
NO	Nitric oxide
OXPHOS	Oxidative phosphorylation
PAMPs	Pathogen-associated molecular patterns
PBMCs	Peripheral blood mononuclear cells
PD	Parkinson’s disease
PDK1	Phosphoinositide-dependent protein kinase 1
PI3K	Phosphatidylinositol 3-kinase
PIP2	Phosphatidylinositol 4,5-bisphosphate
PIP3	Phosphatidylinositol 3,4,5-trisphosphate
PRRs	Pattern-recognition receptors
PSEN 1	Presenilin 1
PSEN 2	Presenilin 2
PYD	Pyrin domain
RLHs	Retinoic acid-inducible gene I-like helicases
ROS	Reactive oxygen species
SAT	Subcutaneous adipose tissue
T2DM	Type 2 diabetes mellitus
TLRs	Toll-like receptors
TNFα	Tumor necrosis factor α
VAT	Visceral adipose tissue
